# Supplementary material for: Resolving the full spectrum of human genome variation using Linked-Reads
Source: Genome Res. 2019 Apr;29(4):635–45. doi: 10.1101/gr.234443.118 (PMC6442396; doi:10.1101/gr.234443.118)
Supplement: Supplemental Material [file supp_gr.234443.118_Supplemental_longranger-2.2.zip › longranger-2.2.tar.bz2!/lr2.2-public/lib/stan/cmdstan/stan/doc/api/html/von__mises_8hpp_source.html]

Stan: src/stan/prob/distributions/univariate/continuous/von\_mises.hpp Source File


|  |  |
| --- | --- |
|  | Stan  2.5.0  probability, sampling & optimization |


- Main Page
- Related Pages
- Namespaces
- Classes
- Files

- File List
- File Members

All Classes Namespaces Files Functions Variables Typedefs Enumerations Enumerator Friends Macros Pages

- src
- stan
- prob
- distributions
- univariate
- continuous

von\_mises.hpp

Go to the documentation of this file.

1 #ifndef STAN\_\_PROB\_\_DISTRIBUTIONS\_\_UNIVARIATE\_\_CONTINUOUS\_\_VON\_MISES\_HPP

2 #define STAN\_\_PROB\_\_DISTRIBUTIONS\_\_UNIVARIATE\_\_CONTINUOUS\_\_VON\_MISES\_HPP

3

4 #include <stan/agrad/partials\_vari.hpp>

5 #include <stan/math/error\_handling.hpp>

6 #include <stan/math.hpp>

7 #include <stan/meta/traits.hpp>

8 #include <stan/prob/constants.hpp>

9 #include <stan/prob/traits.hpp>

10 #include <stan/prob/distributions/univariate/continuous/uniform.hpp>

11

12 namespace stan {

13

14  namespace prob {

15

16  template<bool propto,

17  typename T\_y, typename T\_loc, typename T\_scale>

18  typename return\_type<T\_y,T\_loc,T\_scale>::type

19  von\_mises\_log(T\_y const& y, T\_loc const& mu, T\_scale const& kappa) {

20  static char const\* const function = "stan::prob::von\_mises\_log(%1%)";

21

22  // check if any vectors are zero length

23  if (!(stan::length(y)

24  && stan::length(mu)

25  && stan::length(kappa)))

26  return 0.0;

27

28  using stan::is\_constant\_struct;

29  using stan::math::check\_finite;

30  using stan::math::check\_positive\_finite;

31  using stan::math::check\_greater;

32  using stan::math::check\_nonnegative;

33  using stan::math::check\_consistent\_sizes;

34  using stan::math::value\_of;

35

36  // Result accumulator.

37  double logp = 0.0;

38

39  // Validate arguments.

40  check\_finite(function, y, "Random variable", &logp);

41  check\_finite(function, mu, "Location paramter", &logp);

42  check\_positive\_finite(function, kappa, "Scale parameter", &logp);

43  check\_consistent\_sizes(function, y, mu, kappa, "Random variable",

44  "Location parameter", "Scale parameter",

45  &logp);

46

47  // check if no variables are involved and prop-to

48  if (!include\_summand<propto,T\_y,T\_loc,T\_scale>::value)

49  return logp;

50

51  // Determine constants.

52  const bool y\_const = is\_constant\_struct<T\_y>::value;

53  const bool mu\_const = is\_constant\_struct<T\_loc>::value;

54  const bool kappa\_const = is\_constant\_struct<T\_scale>::value;

55

56  // Determine which expensive computations to perform.

57  const bool compute\_bessel0 = include\_summand<propto,T\_scale>::value;

58  const bool compute\_bessel1 = !kappa\_const;

59  const double TWO\_PI = 2.0 \* stan::math::pi();

60

61  // Wrap scalars into vector views.

62  VectorView<const T\_y> y\_vec(y);

63  VectorView<const T\_loc> mu\_vec(mu);

64  VectorView<const T\_scale> kappa\_vec(kappa);

65

66  DoubleVectorView<true,is\_vector<T\_scale>::value> kappa\_dbl(length(kappa));

67  DoubleVectorView<include\_summand<propto,T\_scale>::value,is\_vector<T\_scale>::value> log\_bessel0(length(kappa));

68  for (size\_t i = 0; i < length(kappa); i++) {

69  kappa\_dbl[i] = value\_of(kappa\_vec[i]);

70  if (include\_summand<propto,T\_scale>::value)

71  log\_bessel0[i] = log(boost::math::cyl\_bessel\_i(0, value\_of(kappa\_vec[i])));

72  }

73  agrad::OperandsAndPartials<T\_y, T\_loc, T\_scale> oap(y, mu, kappa);

74

75  size\_t N = max\_size(y, mu, kappa);

76

77  for (size\_t n = 0; n < N; n++) {

78  // Extract argument values.

79  const double y\_ = value\_of(y\_vec[n]);

80  const double y\_dbl = y\_ - std::floor(y\_ / TWO\_PI) \* TWO\_PI;

81  const double mu\_dbl = value\_of(mu\_vec[n]);

82

83  // Reusable values.

84  double bessel0 = 0;

85  if (compute\_bessel0)

86  bessel0 = boost::math::cyl\_bessel\_i(0, kappa\_dbl[n]);

87  double bessel1 = 0;

88  if (compute\_bessel1)

89  bessel1 = boost::math::cyl\_bessel\_i(-1, kappa\_dbl[n]);

90  const double kappa\_sin = kappa\_dbl[n] \* std::sin(mu\_dbl - y\_dbl);

91  const double kappa\_cos = kappa\_dbl[n] \* std::cos(mu\_dbl - y\_dbl);

92

93  // Log probability.

94  if (include\_summand<propto>::value)

95  logp -= LOG\_TWO\_PI;

96  if (include\_summand<propto,T\_scale>::value)

97  logp -= log\_bessel0[n];

98  if (include\_summand<propto,T\_y,T\_loc,T\_scale>::value)

99  logp += kappa\_cos;

100

101  // Gradient.

102  if (!y\_const)

103  oap.d\_x1[n] += kappa\_sin;

104  if (!mu\_const)

105  oap.d\_x2[n] -= kappa\_sin;

106  if (!kappa\_const)

107  oap.d\_x3[n] += kappa\_cos / kappa\_dbl[n] - bessel1 / bessel0;

108  }

109

110  return oap.to\_var(logp);

111  }

112

113  template<typename T\_y, typename T\_loc, typename T\_scale>

114  inline typename return\_type<T\_y,T\_loc,T\_scale>::type

115  von\_mises\_log(T\_y const& y, T\_loc const& mu, T\_scale const& kappa) {

116  return von\_mises\_log<false>(y, mu, kappa);

117  }

118

119  // The algorithm used in von\_mises\_rng is a modified version of the

120  // algorithm in:

121  //

122  // Efficient Simulation of the von Mises Distribution

123  // D. J. Best and N. I. Fisher

124  // Journal of the Royal Statistical Society. Series C (Applied Statistics), Vol. 28, No. 2 (1979), pp. 152-157

125  //

126  // See licenses/stan-license.txt for Stan license.

127

128  template <class RNG>

129  inline double

130  von\_mises\_rng(const double mu,

131  const double kappa,

132  RNG& rng) {

133  using boost::variate\_generator;

134  using stan::prob::uniform\_rng;

135

136  static const char\* function = "stan::prob::von\_mises\_rng(%1%)";

137

138  stan::math::check\_finite(function,mu,"mean",(double\*)0);

139  stan::math::check\_positive\_finite(function,kappa,"inverse of variance",

140  (double\*)0);

141

142  double r = 1 + pow((1 + 4 \* kappa \* kappa), 0.5);

143  double rho = 0.5 \* (r - pow(2 \* r, 0.5)) / kappa;

144  double s = 0.5 \* (1 + rho \* rho) / rho;

145

146  bool done = 0;

147  double W;

148  while (!done) {

149  double Z = std::cos(stan::math::pi() \* uniform\_rng(0.0, 1.0, rng));

150  W = (1 + s \* Z) / (s + Z);

151  double Y = kappa \* (s - W);

152  double U2 = uniform\_rng(0.0, 1.0, rng);

153  done = Y \* (2 - Y) - U2 > 0;

154

155  if(!done)

156  done = log(Y / U2) + 1 - Y >= 0;

157  }

158

159  double U3 = uniform\_rng(0.0, 1.0, rng) - 0.5;

160  double sign = ((U3 >= 0) - (U3 <= 0));

161

162  // it's really an fmod() with a positivity constraint

163  return sign \* std::acos(W) + fmod(fmod(mu,2\*stan::math::pi())+2\*stan::math::pi(),2\*stan::math::pi());

164  }

165

166  }

167 }

168 #endif

stan::agrad::sin

fvar< T > sin(const fvar< T > &x)

**Definition:** sin.hpp:14

stan::math::check\_greater

bool check\_greater(const char \*function, const T\_y &y, const T\_low &low, const char \*name, T\_result \*result)

**Definition:** check\_greater.hpp:58

traits.hpp

stan::prob::von\_mises\_rng

double von\_mises\_rng(const double mu, const double kappa, RNG &rng)

**Definition:** von\_mises.hpp:130

stan::agrad::acos

fvar< T > acos(const fvar< T > &x)

**Definition:** acos.hpp:16

stan::agrad::OperandsAndPartials::to\_var

T\_return\_type to\_var(double logp)

**Definition:** partials\_vari.hpp:138

stan::math::check\_positive\_finite

bool check\_positive\_finite(const char \*function, const T\_y &y, const char \*name, T\_result \*result)

**Definition:** check\_positive\_finite.hpp:12

uniform.hpp

stan::math::sign

int sign(const T &z)

**Definition:** sign.hpp:9

stan::agrad::pow

fvar< T > pow(const fvar< T > &x1, const fvar< T > &x2)

**Definition:** pow.hpp:17

error\_handling.hpp

stan::agrad::floor

fvar< T > floor(const fvar< T > &x)

**Definition:** floor.hpp:15

stan::length

size\_t length(const T &)

**Definition:** traits.hpp:159

stan::DoubleVectorView

DoubleVectorView allocates double values to be used as intermediate values.

**Definition:** traits.hpp:358

stan::math::check\_finite

bool check\_finite(const char \*function, const T\_y &y, const char \*name, T\_result \*result)

Checks if the variable y is finite.

**Definition:** check\_finite.hpp:52

partials\_vari.hpp

stan::agrad::value\_of

T value\_of(const fvar< T > &v)

Return the value of the specified variable.

**Definition:** value\_of.hpp:16

constants.hpp

stan::is\_vector

**Definition:** traits.hpp:83

stan::agrad::OperandsAndPartials

A variable implementation that stores operands and derivatives with respect to the variable...

**Definition:** partials\_vari.hpp:76

traits.hpp

stan::return\_type::type

boost::math::tools::promote\_args< typename scalar\_type< T1 >::type, typename scalar\_type< T2 >::type, typename scalar\_type< T3 >::type, typename scalar\_type< T4 >::type, typename scalar\_type< T5 >::type, typename scalar\_type< T6 >::type >::type type

**Definition:** traits.hpp:406

stan::is\_constant\_struct

Metaprogram to determine if a type has a base scalar type that can be assigned to type double...

**Definition:** traits.hpp:57

stan::math::value\_of

double value\_of(const T x)

Return the value of the specified scalar argument converted to a double value.

**Definition:** value\_of.hpp:24

stan::prob::include\_summand

Template metaprogram to calculate whether a summand needs to be included in a proportional (log) prob...

**Definition:** traits.hpp:35

math.hpp

stan::agrad::OperandsAndPartials::d\_x2

VectorView< double \*, is\_vector< T2 >::value, is\_constant\_struct< T2 >::value > d\_x2

**Definition:** partials\_vari.hpp:83

stan::prob::uniform\_rng

double uniform\_rng(const double alpha, const double beta, RNG &rng)

**Definition:** uniform.hpp:339

stan::math::check\_nonnegative

bool check\_nonnegative(const char \*function, const T\_y &y, const char \*name, T\_result \*result)

**Definition:** check\_nonnegative.hpp:52

stan::math::check\_consistent\_sizes

bool check\_consistent\_sizes(const char \*function, const T1 &x1, const T2 &x2, const char \*name1, const char \*name2, T\_result \*result)

**Definition:** check\_consistent\_sizes.hpp:13

stan::max\_size

size\_t max\_size(const T1 &x1, const T2 &x2)

**Definition:** traits.hpp:191

stan::agrad::cos

fvar< T > cos(const fvar< T > &x)

**Definition:** cos.hpp:15

stan::prob::von\_mises\_log

return\_type< T\_y, T\_loc, T\_scale >::type von\_mises\_log(T\_y const &y, T\_loc const &mu, T\_scale const &kappa)

**Definition:** von\_mises.hpp:19

stan::agrad::fmod

fvar< T > fmod(const fvar< T > &x1, const fvar< T > &x2)

**Definition:** fmod.hpp:16

stan::agrad::OperandsAndPartials::d\_x1

VectorView< double \*, is\_vector< T1 >::value, is\_constant\_struct< T1 >::value > d\_x1

**Definition:** partials\_vari.hpp:82

stan::agrad::OperandsAndPartials::d\_x3

VectorView< double \*, is\_vector< T3 >::value, is\_constant\_struct< T3 >::value > d\_x3

**Definition:** partials\_vari.hpp:84

stan::math::pi

double pi()

Return the value of pi.

**Definition:** constants.hpp:77

stan::agrad::log

fvar< T > log(const fvar< T > &x)

**Definition:** log.hpp:15

stan::VectorView

VectorView is a template metaprogram that takes its argument and allows it to be used like a vector...

**Definition:** traits.hpp:275


---

[ Stan Home Page ]

*© 2011–2014,
Stan Development Team.*
